# Two separate Ni^2+^-sensitive voltage-gated Ca^2+^channels modulate transretinal signalling in the isolated murine retina

**DOI:** 10.1111/j.1755-3768.2011.02167.x

**Published:** 2011-11

**Authors:** Maged Alnawaiseh, Walid Albanna, Chien-Chang Chen, Kevin P Campbell, Jürgen Hescheler, Matthias Lüke, Toni Schneider

**Affiliations:** 1 Institute of Neurophysiology, University of CologneKoeln, Germany; 2 Molecular Physiology and Biophysics, The University of Iowa College of MedicineIowa City, Iowa, USA; 3 Departments of Molecular Physiology and Biophysics, Neurology, and Internal Medicine, and the Howard Hughes Medical Institute, University of Iowa Roy J. and Lucille A. Carver College of MedicineIowa City, Iowa, USA; 4 Center of Molecular Medicine Cologne (CMMC), University of CologneKoeln, Germany; 5 University Eye Hospital, University of LübeckRatzeburger Allee 160, Lübeck, Germany

**Keywords:** adaptation, feedback control, isolated retina, pharmacoresistant, R-type, T-type

## Abstract

**Purpose:**

Light-evoked responses from vertebrate retinas were recorded as an electroretinogram (ERG). The b-wave is the most prominent component of the ERG, and in the bovine retina its NiCl_2_-sensitive component was attributed to reciprocal signalling by pharmacoresistant R-type voltage-gated Ca^2+^ channels, which similar to other voltage-dependent Ca^2+^ channels trigger and control neurotransmitter release. The murine retina has the great advantage that the effect of gene inactivation for Ni^2+^-sensitive Ca^2+^ channels can be analysed to prove or disprove that any of these Ca^2+^ channels is involved in retinal signalling.

**Methods:**

Superfused retinas from different murine genotypes lacking either one or both highly Ni^2+^-sensitive voltage-gated Ca^2+^ channels were used to record their *ex vivo* ERGs.

**Results:**

The isolated retinas from mice lacking Ca_v_2.3 R-type or Ca_v_3.2 T-type or both voltage-gated Ca^2+^ channels were superfused with a NiCl_2_ (15 μm) containing nutrient solution. The change in the b-wave amplitude and implicit time, caused by NiCl_2_, was calculated as a difference spectrum and compared to data from control animals. From the results, it can be deduced that Ca_v_2.3 contributes rather to a later component in the b-wave response, while in the absence of Ca_v_3.2 the gain of Ni^2+^-mediated increase in the b-wave amplitude is significantly increased, probably due to a loss of reciprocal inhibition to photoreceptors. Thus, each of the Ni^2+^-sensitive Ca^2+^ channels contributes to specific features of the b-wave response.

**Conclusion:**

Both high-affinity Ni^2+^-sensitive Ca^2+^ channels contribute to transretinal signalling. Based on the results from the double knockout mice, additional targets for NiCl_2_ must contribute to transretinal signalling, which will be most important for the structurally similar physiologically more important heavy metal cation Zn^2+^.

## Introduction

Under the various light conditions, the processing of visual information originates from rods and cones. Rod photoreceptors reliably signal the capture of a single photon, whereas cones require higher rates of quantum capture to cause a significant change in glutamate release from the dark. Consecutively, the visual system continuously adjusts its sensitivity to the conditions of the immediate environment. GABAergic feedback inhibition may offer a mechanism to be involved in dark and light adaptation. It shapes visual signalling in the inner retina (Chavez et al. 2010) and was identified in the bovine retina to be initiated by pharmacoresistant but Ni^2+^-sensitive voltage-gated Ca^2+^ channels (Siapich et al. 2009). In the present report, the murine retina was isolated from different Ca^2+^ channel–deficient mouse lines to investigate the role of two high-affinity Ni^2+^-sensitive Ca^2+^ channels during feedback control of the b-wave response.

At the photoreceptor level, the influx of Ca^2+^ through L-type voltage-gated Ca^2+^ channels triggers the Ca^2+^-dependent release of synaptic vesicles (Morgans et al. 2001), containing the neurotransmitter glutamate (for review see Barnes & Kelly 2002; Heidelberger et al. 2005). At the level of bipolar and higher-order neurons, additional voltage-gated Ca^2+^ channel types may contribute to the specific kinetics of visual processing and adaptation. Intense immunoreactivity for Ca_v_2.1 (P/Q-type) and Ca_v_2.2 (N-type) was observed in both the outer and inner plexiform layers. In addition, Ca_v_2.2-specific immunoreactivity was found in the outer and inner nuclear layers. But staining for Ca_v_2.3 (R-type) Ca^2+^ channels was rather widely distributed in all three nuclear layers and in the inner plexiform layer (Xu et al. 2002). Based on electrophysiological criteria, bipolar neurons can be distinguished in those with prominent L-type Ca^2+^ currents (L-rich) and those expressing various T-type Ca^2+^ currents (T-rich cone bipolar cells) (Pan et al. 2001; Hu et al. 2009).

Secondary- and tertiary-order neurons also respond with graded and relatively small changes in their membrane potential, which leads to the most prominent component of the electroretinogram (ERG), the b-wave. ON-centre bipolar and horizontal cells are believed to be the major contributors to the b-wave in addition to third-order neurons as amacrine cells (for review of literature see Dong & Hare 2002). Horizontal cells are the first interneurons from the retinal network that participate in lateral information processing in the vertebrate retina. As they do not generate sodium-dependent action potentials, Ca^2+^ channel regulation in horizontal cells may be important for shaping graded potentials derived from photoreceptors and transferred to bipolar neurons (Schubert et al. 2006).

Related to their molecular structure, the heteromultimeric voltage-gated Ca^2+^ channels consist of an ion-conducting α1 subunit and a set of auxiliary subunits (for review see: (Catterall 2000; Perez-Reyes 2003; Lacinova 2005; Striessnig & Koschak 2008)). They are subdivided into L-type (Ca_v_1), non-L-type (Ca_v_2) and T-type (Ca_v_3) voltage-gated Ca^2+^ channels, according to their electrophysiological and pharmacological properties. P/Q-, N- and E/R-type channels are members of the non-L-type channels, and their α1 subunits are addressed as Ca_v_2.1, Ca_v_2.2 and Ca_v_2.3, respectively (Kamp et al. 2005). Transcripts of at least half of the voltage-gated Ca^2+^ channels of the CNS were detected in the retina, and dihydropyridine-sensitive L-type, N- and P/Q-type as well as blocker-insensitive T-type voltage-gated Ca^2+^ channels were referred to visual signal transduction so far (Kamphuis & Hendriksen 1998; Pan et al. 2001; Heidelberger et al. 2005). Low-voltage-activated T-type Ca^2+^ currents have been recorded from the bipolar cells of the rat retina (Pan 2000; Pan et al. 2001). Particularly, L-type voltage-gated Ca^2+^ channels have been well analysed in the retina (Morgans et al. 2001), as mutations in Ca_v_1.4 (α1F) and in the auxiliary β_2_-subunit cause the loss of visual sensitivity (Ball & Gregg 2002), and mutations in Ca_v_1.4 itself are responsible for the incomplete form of X-linked congenital stationary blindness (Bech-Hansen et al. 1998; Strom et al. 1998; McRory et al. 2004), but also mutations in auxiliary subunits cause major damage in the retina (Wycisk et al. 2006).

The present study was initially inspired by results from isolated frog retina, in which the perfusion with NiCl_2_ caused biphasic effects. While low NiCl_2_ concentrations (up to 30 μm) stimulated the b-wave response, high concentrations (100 μm) reduced the b-wave response (Sickel 1972a). This Ni^2+^-effect was consecutively evaluated on the ERG of higher vertebrates as the bovine retina leading to the detection of a similar transient stimulation and to a putative role for Ca_v_2.3 R-type voltage-gated Ca^2+^ channels in bovine retinal signalling (Lüke et al. 2005a). But among voltage-gated Ca^2+^ channels, besides Ca_v_2.3/R-type another highly Ni^2+^-sensitive voltage-gated Ca^2+^ channel (Ca_v_3.2/T-type) is known to modulate neuronal signal transduction. Therefore, we switched to investigations of the murine retina. For both Ca^2+^ channel types (Ca_v_2.3 and Ca_v_3.2), nonlethal mouse lines are available (Pereverzev et al. 2002; Chen et al. 2003) and were used in the present study, either as a separate mouse line or after inbreeding to generate a double knockout mouse line, lacking Ca_v_2.3 and Ca_v_3.2. The method of a stable and longer-lasting ERG recording was recently transferred successfully from the bovine to the murine retina (Albanna et al. 2009), which was a prerequisite for the present study. The results demonstrate that reciprocal signal transduction to retinal bipolar neurons may be a mechanism in visual adaptation, which should occur via two Ni^2+^-sensitive voltage-gated Ca^2+^ channels.

## Materials and Methods

### Materials

Glucose and the constituents of the nutrient solution used for retinal superfusion were purchased from Merck (p.a. grade). All stock solutions for the nutrient solutions were sealed in glass tubes (Gerresheimer Wertheim GmbH, Wertheim, Germany) to establish reproducibility of ERG recordings. Deionized water (<0.1 μS/cm) was additionally glass distilled and autoclaved in glass bottles. All solutions were prepared in autoclaved glass bottles, using sterile distilled water.

For the preparation of the murine retina (Albanna et al. 2009), a superfine scissor (WPI, Nr. 501839) and ultrafine suturing forceps were used (WPI, Nr. 555063FT). Further, a 27-gauge needle (Sterican, size 20: 0.4 mm × 20 mm Bl/LB) was used to punch a hole into the cornea of the extirpated eye bulb.

### Animals

Murine retinas were isolated from mice of our animal facility department, in which the light–dark regime was 12:12 hr, and the light intensity between 5 and 10 lx at the surface of the animal cages. The mice were preincubated for dark adaptation overnight.

Inbred strains on a C57Bl/6 background were used for retina isolation of Ca_v_2.3- (Pereverzev et al. 2002) and Ca_v_3.2-deficient mice (Chen et al. 2003). Double knockout mice, which were lacking both the mentioned highly Ni^2+^-sensitive Ca^2+^channels, were generated from homozygous mice deficient for one gene by further inbreeding.

DNA-containing tissue samples were collected from tail biopsies. DNA was extracted and used as template for genotyping. Transcripts of Ca_v_2.3 were detected by RT-PCR using primers, which flanked the deleted exon 2 and exon 3 region (Pereverzev et al. 2002). For Ca_v_2.3, mouse lines were used as separate inbred strains for Cav2.3(+|+) and Cav2.3(−|−), each after the fourth backcrossing in C57Bl/6 mice. For Cav3.2, mouse lines were used in pure C57Bl/6 background. For the experiments, we used littermates, and genotyped by RT-PCR.

The German Animal Experiments Inspectorate approved the treatment of mice involved in the experiments and also approved the animal-housing facilities. We have considered the ethical aspects of the study and followed the guidelines of the European Communities Council Directive of 24 November 1986 (86/609/EEC).

### Preparation of the retina and incubation conditions

**Fig. 1:**
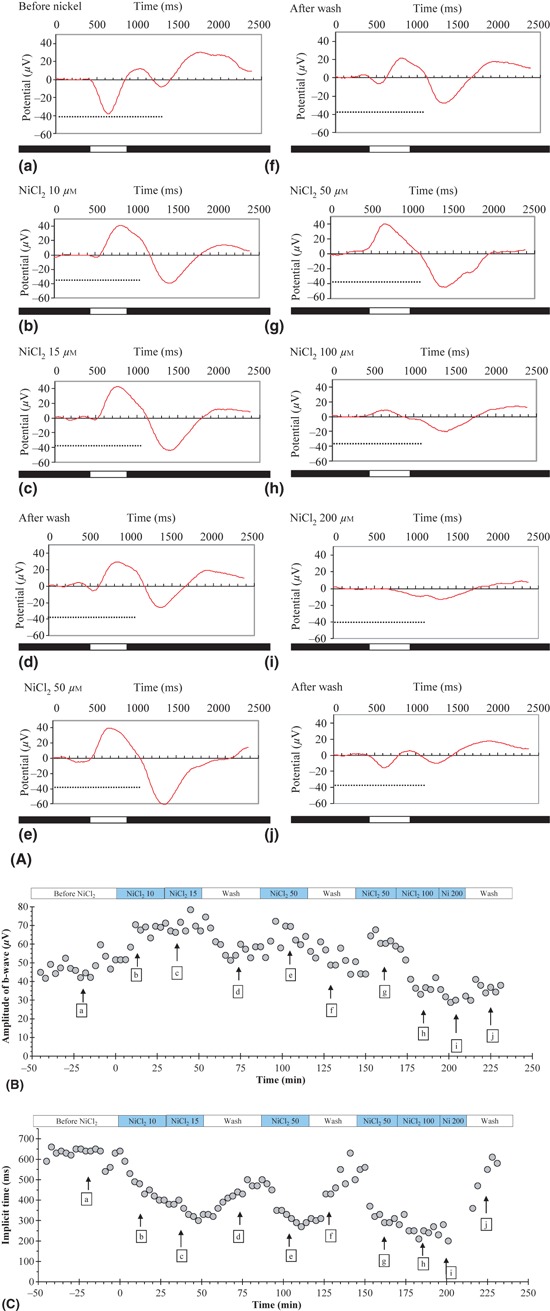
**(A).** Dual effects of NiCl_2_ on the murine electroretinogram (ERG) b-wave. ERG recording (preincubation protocol for 4 hr, see Materials and Methods) and plot of the b-wave amplitude (panel B) and implicit time (panel C) after flashes of light (0.5 seconds, 63 mlx). Light-evoked responses were recorded every 3 min. Superfusion of nutrient solution at a flow rate of 2 ml/min. Small letters in panel (B) indicate individual ERG traces, which are shown in panel (A). (A) Individual ERG recordings from the time points as indicated in panel (B) and (C). Note, the black-and-white bar under each ERG denotes the time when the light flash occurs. (B) After reaching equilibrium of a stable b-wave amplitude, NiCl_2_ was added as indicated to the perfusing solution at 10 and subsequently at 15 μm, which was washed out. At 50 μm NiCl_2_, a lower and transient increase in the b-wave amplitude was observed, which after another washout was finally inhibited at 100 and 200 μm NiCl_2_. (C) Plot over time of the implicit times from the ERGs shown in panel (B).

Murine eyes were enucleated immediately post-mortem from mice at the age of 15–25 weeks. The enucleated eyes were transferred in darkness into a serum-free, oxygen-saturated standard medium (‘nutrient solution’) containing 120 mm NaCl, 2 mm KCl, 0.1 mm MgCl_2_, 0.15 mm CaCl_2_, 1.5 mm NaH_2_PO_4_, 13.5 mm Na_2_HPO_4_ and 5 mm glucose. The resulting pH was 7.8. The bathing solution was empirically optimized (Sickel 1972b). Subsequently, the isolation of the murine retina was started immediately and carried out under dim red light. Details are visualized in a video animation, which is accessible via the supplement in a recent report (Albanna et al. 2009). Initial experiments (see Fig. 1 and Table 1) were performed with retina segments as shown in the animation. Later on, full retinas were used for ERG recordings, and the 4-h preincubation period was omitted and replaced by about 30-min equilibration period, as mentioned in the result section.

**Table 1:** Effect of NiCl_2_ on retinal b-wave amplitude from Ca_v_2.3-deficient and control mice. Retina segments were isolated either from controls (panel A.) or from Ca_v_2.3-deficient mice (panel B.; isolation of retinas according to Albanna et al., 2009). Abbreviation: n.d. = not determined; p-value < 0.05 reveals significant differences between control and Ca_v_2.3-deficient mice

					Amplitude of b-wave, μV					Comparison of ratios:		
						
							After adding	After adding	After	Ratio of		
										
							NiCl_2_	NiCl_2_				
												
					Before application (= NLsg)		10 or 15 μM	30 μM	Washout (= NLsg)	column # 2/# 1	column # 3/# 1	column #3/#4 or #2/#4
								
Exp. #	Experimental design (dosis of NiCl_2_ in μM)				Column-#:	1	2	3	4	5	6	7
(A) Ca_v_2.3+/+ mice												
	1	2	3	4	Mean	6.15	8.51	11.18	6.87	**1.38**	**1.82**	**1.63**
1	NLsg-	(15 NiCl_2_)-	(30 NiCl_2_)-	NLsg								
					SEM	0.33	0.17	0.19	0.17			
	1	2	3	4	*n*	6	6	6	6			
2	NLsg-	(15 NiCl_2_)-	(30 NiCl_2_)-	NLsg	Mean	14.88	14.93	16.63	11.58	**1.00**	**1.12**	**1.44**
					SEM	0.22	0.43	0.58	1.19			
	1	2	3	4	*n*	4	4	6	4			
3	NLsg-	(15 NiCl_2_)-	(30 NiCl_2_)-	NLsg	Mean	4.97	7.18	7.63	4.65	**1.44**	**1.54**	**1.64**
					SEM	0.11	0.34	0.66	0.75			
	1		3	4	*n*	6	6	6	4			
4	NLsg-		(30 NiCl_2_)-	NLsg	Mean	9.7	n.d.	16.2	9.28		**1.67**	**1.75**
					SEM	0.09		0.42	0.47			
	1		3	4	*n*	6		6	6			
5	NLsg-		(30 NiCl_2_)-	NLsg	Mean	5.9	n.d.	9.0	6.23		**1.53**	**1.44**
					SEM	0.14		0.21	0.30			
	1		3	4	*n*	6		6	6			
6	NLsg-		(30 NiCl_2_)-	NLsg	Mean	9.82	n.d.	13.91	10.85		**1.42**	**1.28**
					SEM	0.08		0.36	0.07			
	1	2	3	4	*n*	4		4	7			
7	NLsg-	(**10** NiCl_2_)-	(30 NiCl_2_)-	NLsg	Mean	6.55	6.99	8.39	7.44	**1.07**	**1.28**	**1.13**
					SEM	0.37	0.28	0.25	0.36			
	1	2	3	4	*n*	4		4	7			
8	NLsg-	(15 NiCl_2_)-	(30 NiCl_2_)-	NLsg	Mean	24.98	30.53	20.27	22.72	**1.22**	**0.81**	**0.89**
					SEM	0.62	0.49	0.18	0.20			
	1	2		4	*n*	6	6	6	6			
9	NLsg-	(**10** NiCl_2_)-		NLsg	Mean	12.3	23.50	n.d.	14.10	**1.91**		**1.67**
					SEM	0.60	1.20		1.20			
	1	2	3	4	*n*	6	6		6			
10	NLsg-	(**10** NiCl_2_)-	(30 NiCl2)-	NLsg	Mean	98.3	136.44	142.8	111.2	**1.39**	**1.45**	**1.23**
					SEM	5.20	2.20	3.6	2.80			
					*n*	6	6	6	6			
	Mean									**1.35**	**1.40**	**1.41**
	SD									0.30	0.30	0.27
	SEM									**0.11**	**0.10**	**0.09**
	*n*									7	9	10
	Column-#					1	2	3	4	5	6	7
(B) Ca_v_2.3−/− mice												
	1		3	4								
1	NLsg-		(30 NiCl_2_)-	NLsg	Mean	5.41	n.d.	4.29	4.52		**0.79**	**0.95**
					SEM	0.40		0.41	0.44			
	1		3	4	*n*	6		6	6			
2	NLsg-		(30 NiCl_2_)-	NLsg	Mean	3.86	n.d.	3.28	3.86		**0.85**	**0.85**
					SEM	0.48		0.38	0.15			
	1		3	4	*n*	8		8	8			
3	NLsg-		(30 NiCl_2_)-	NLsg	Mean	2.91	n.d.	4.72	3.37		**1.62**	**1.40**
					SEM	0.51		0.16	0.26			
	1	2		4	*n*	8			8	5		
4	NLsg-	(**10** NiCl_2_)		-NLsg	Mean	6.7	6.7	n.d.	6.5	**1.00**		**1.03**
					SEM	0.21	0.14		0.26			
	1	2		4	*n*	8	8		6			
5	NLsg-	(**10** NiCl_2_)		-NLsg	Mean	4.4	4.8	n.d.	3.7	**1.10**		**1.31**
					SEM	0.21	0.17		0.12			
					*n*	8	8		8			
6	NLsg-	(**15** NiCl_2_)-	NLsg		Mean	16.21	17.59	n.d.	14.59	**1.09**		**1.21**
					SEM	0.65	0.31		0.73			
	1	3	4		*n*	6	3		5			
7	NLsg-	(30 NiCl_2_)-	NLsg		Mean	18.28	n.d.	20.45	18.03		**1.12**	**1.13**
					SEM	0.36		1.21	0.50			
					*n*	11		11	6			
	Mean									**1.06**	**1.10**	**1.12**
	SD									0.05	0.38	0.20
	SEM									**0.03**	**0.19**	**0.07**
	*n*									3	4	7
	Student *t*-test, p value									**0.152**	**0.143**	**0.034**

A. The data from 10 independent experiments of control animals (retina segments from 6 different mice) are summarized in individual lines after applying either 10, 15 or 30 μM NiCl_2_ to the superfusing nutrient solution (= NLsg), as indicated in the column ‘experimental design’. The columns with data are numbered and contain the mean values from consecutive ERGs (*n* = number of consecutive ERGs) as the amplitude of the b-wave either after reaching equilibrium (“Before application”, column 1), or after adding NiCl_2_ as indicated in column 2 and 3. To minimize the effect of a routinely observed slow run-up, we also calculated the b-wave amplitude after washout in column 4. In column 5 (for 10 and 15 μM NiCl_2_) and 6 (for 30 μM NiCl_2_), the ratio of a NiCl_2_-mediated change of b-wave amplitude was calculated. In column 7 we controlled for reversibility by calculating the ratio of b-wave under NiCl_2_ divided by the amplitude after wash (either column #3/#4 or #2/#4).

B. Similar as in panel A., the data from 7 independent experiments of Ca_v_2.3-deficient mice (retina segments from 4 different mice) are summarized.

Enucleated murine eyes were opened from the front side first by punching a tiny hole through the cornea to relieve the aqueous humour from the anterior and posterior chamber of the eye and thus to reduce the tension. Thereafter, the cornea was removed, and a tiny triangle-shaped incision was made into the sclera for later separation of the proximal eye cup, and for the immediate introduction of small forceps. Iris and lens were carefully removed. Consecutively, the opened eye cup was held by forceps to detach the retina from the pigment epithelium by repetitive moving in the nutrient solution. The successive separation of the retina from the pigment epithelium has to be performed gently and is supported by the cutting of the sclera layers from outside.

Following dissection from the underlying pigment epithelium, the retina was mounted on a plastic mesh occupying the centre of the perfusing chamber. The retina segment adheres by itself, and the supporting ring is turned upside down to move the retina-loaded ring into the holder of the recording chamber. The recording chamber, which is otherwise identical to the former chamber for recording of bovine retina segments (Sickel 1965), is locked by two gaskets and two glasses, one on each side, and finally by a brass metal screw surrounding the inner part of the recording chamber. Thereafter, the nutrient solution is perfused over the front and the rear side of the closed recording chamber.

The ERG was recorded *via* two silver/silver chloride electrodes on either side of the retina with the recording chamber containing the retina placed in an electrically and optically isolated air thermostat. The recording chamber was continuously perfused at a perfusion velocity of 2 ml/min, controlled by a roller pump. Temperature was kept constant at 27.5°C. To avoid hypoxic conditions during the preparation of the isolated retinas as well as during the perfusion, the nutrient standard solution was pre-equilibrated and continuously saturated with oxygen and sometimes monitored by a Clark oxygen electrode during the experiments. From the dark-adapted retina, ERGs in response to a single white flash were recorded at intervals of 3 min. Equilibrium was reached between 10 and 30 min of perfusion for the murine retina. The duration of light stimulation was 500 ms controlled by a timer operating a mechanical shutter system. The prestimulus delay was 150, 250 or 350 ms as indicated by the stimulus bar in each figure. The flash intensity was set to 63 mlx at the retinal surface using calibrated neutral-density filters. The ERG was amplified and bandpass-limited between 1 and 300 Hz. The signal was AD converted and stored using a PC-based signal acquisition and analysis system (DASY-Lab).

For each experiment, an unused retina segment or full retina was transferred to the recording chamber. The retina was superfused with the nutrient solution and stimulated repetitively until stable responses were recorded. Switching from one solution to another was performed without compensation for the latency of drug delivery, which can be seen in the labelled bars in each figure.

### Data analysis

The a-wave amplitude was measured from the zero line to the consecutive trough. The b-wave amplitude was measured from the trough of the a-wave to the peak of the b-wave, as indicated. For the statistical analysis of the a- and b-wave amplitude, the software programs ‘Origin 6.0’ (Microcal) and ‘Excel 2003’ (Microsoft) were used. ERG traces were generated from central moving average values, for which the unweighted mean of 13 data points was determined (from six previous to six future data points).

Significance was calculated with Student’s *t*-test. Levels of p ≤ 0.05 were considered as statistically significant (*) and p ≤ 0.01 as highly significant (**).

## Results

### Effect of NiCl_2_ concentrations on the retinal b-wave

Recording bovine retinal signalling led to a full ERG response after a 60- to 90-min adaptation period (Lüke et al. 2005b). After reaching an equilibrium for light-evoked responses, bovine retina could be further stimulated by NiCl_2_ (15 μm) up to 1.5-fold (Lüke et al. 2005a). However, we also observed that the Ni^2+^-mediated stimulation of the ERG b-wave amplitude was less stable over time than the basic retinal signalling (Siapich et al. 2009). Therefore, even for bovine ERG recordings we used either fast protocols without adaptation or included an adaptation period.

For the isolated murine retina, we did both. Later, we skipped extended adaptations, but initially, we used a 4-hr adaptation period in darkness at room temperature, before we started to record light evoked responses as an ERG with a prominent b-wave, as soon as it was superfused with the oxygenized nutrition solution. The recording protocol, which was initially empirically optimized for bovine ERG recording, was recently modified and adapted to mice by using a tenfold higher stimulus light intensity (63 mlx instead of 6.3 mlx) and a temperature of only 27.5°C instead of 30°C (Albanna et al. 2009). Under these conditions, similar increases in the ERG b-wave amplitude were observed at 10 and 15 μm NiCl_2_ (Fig. 1A,B) as known for the bovine retina but at lower light intensity (Lüke et al. 2005a; Siapich et al. 2009). Finally at 15 μm NiCl_2_, the b-wave amplitude increased significantly 1.44-fold from initially 48.6 ± 2.2 μV (*n* = 8 consecutive recordings) to 69.8 ±1.4 μV (*n* = 8), which was partially reversed to 55.7 ± 1.2 μV (*n* = 8) after washout of NiCl_2_. During this procedure, the implicit time was severely shortened (Fig. 1C). The shift of the implicit time results from a strong reduction in the apparent a-wave, which is overrun by the increasing b-wave and may cause the shifts of implicit times (Fig. 1A-a–A-c), which were absent in bovine ERG recordings with no visible a-wave component. The increase in the NiCl_2_ concentration to 50 μm caused only a minor and transient increase, and finally at 100 and 200 μm only an inhibition of the b-wave amplitude (Fig. 1B). In a total of 10 experiments with control animals, reversible NiCl_2_-mediated b-wave increases were quantified, leading as a mean to a 1.4-fold stimulation of the b-wave amplitude (Table 1). In a similar set of seven experiments with Ca_v_2.3-deficient mice, we observed a 1.1- ± 0.2-fold increase, which did not differ significantly from controls (Table 1B). If we quantified the ratio of Ni^2+^-stimulation compared to the washout, the 1.12-fold increase (±0.07; *n* = 7 different experiments) in Ca_v_2.3-deficient mice was a statistically significant (p = 0.034) difference from the controls of a 1.41-fold increase (±0.1; *n* = 10 different experiments; Table 1A), leading to the assumption that at least part of the Ni^2+^-mediated increase in the b-wave may be mediated through Ca_v_2.3-triggered neurotransmitter release, as suggested for bovine retina (Siapich et al. 2009).

**Fig. 2:**
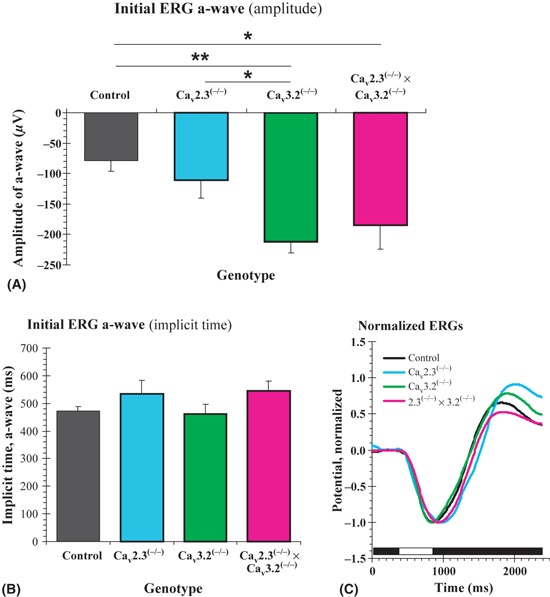
Analysis and comparison of initial electroretinogram (ERG) signals of all four murine genotypes before adding NiCl_2_. (A) Initial ERG recording before adding NiCl_2_ were dominated by the presence of a-waves with different amplitudes, which are plotted for each genotype. Mean values were taken from 4 to 6 individual retinas from each genotype and are presented with SEM values. (B) Implicit times were plotted as mean values plus SEM (*n* = 4–6) for each genotype. (C) Normalized current traces were calculated from mean values and are superimposed for all genotypes.

**Fig. 3:**
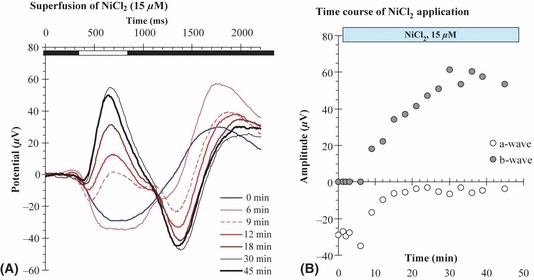
Increase in b-wave amplitude at low (15 μm) NiCl_2_ after only a short pre-equilibration (30 min) period. Individual electroretinogram recordings from control mice at the indicated time intervals were superimposed (panel A), and the b-wave amplitude was plotted versus time after adding 15 μm NiCl_2_ to the nutrient solution (panel B). Note, that maximal amplitude of the b-wave is reached after 30 min of superfusion, while the apparent a-wave is reduced dramatically.

The mentioned prolonged preincubation period of 4 hr represented a risk in the sense that we did not record a NiCl_2_-mediated stimulation in the retinas from control mice in more than 50% of the experiments. Therefore, we shifted the isolation protocol to a version with no prolonged preincubation period, taking into account lower initial b-wave amplitudes. The freshly isolated retina segments were immediately placed into a recording chamber, and the recording protocol was started to reach a rather stable incomplete ERG with no (Fig. 2) or only a minor b-wave (Fig. 3A, 0 min). Such an a-wave-dominated ERG was recorded after 30 min of superfusion and was compared for all four genotypes before adding any substances. Control retinas (*n* = 5) yielded a-waves with an amplitude of −79 ± 17 μV (Fig. 2A). The a-wave amplitudes of Ca_v_2.3-deficient mice (*n* = 6) did not differ significantly from controls with −111 ± 29 μV (p = 0.40). However, after inactivation of Ca_v_3.2 (*n* = 4 retinas) and after inactivation of both highly Ni^2+^-sensitive Ca^2+^ channels (*n* = 6), a-wave amplitude was increased significantly to −212 ± 18 μV (p = 0.0011) and −184 ± 39 μV (p = 0.049), respectively, suggesting that Ca_v_3.2 rather than Ca_v_2.3 may be involved in reciprocal signalling to photoreceptors. No significant differences were found for implicit times between any of the four genotypes (Fig. 2B). Even normalized traces from all four genotypes revealed similar kinetics for the corresponding photoreceptor responses (Fig. 2C).

To record the development of a full, b-wave-dominated ERG, we calculated a mean ERG out of four consecutive ERG traces within 12 min and started the NiCl_2_ (15 μm) superfusion (Fig. 3B). After 6–9 min (Fig. 3A), the b-wave amplitude started to rise to maximal amplitudes, which it reached after about 24 min (Fig. 3B) and stayed stable within the remaining recording time (up to 45 min). The stimulatory effect of NiCl_2_ could only partially be washed out.

**Fig. 4:**
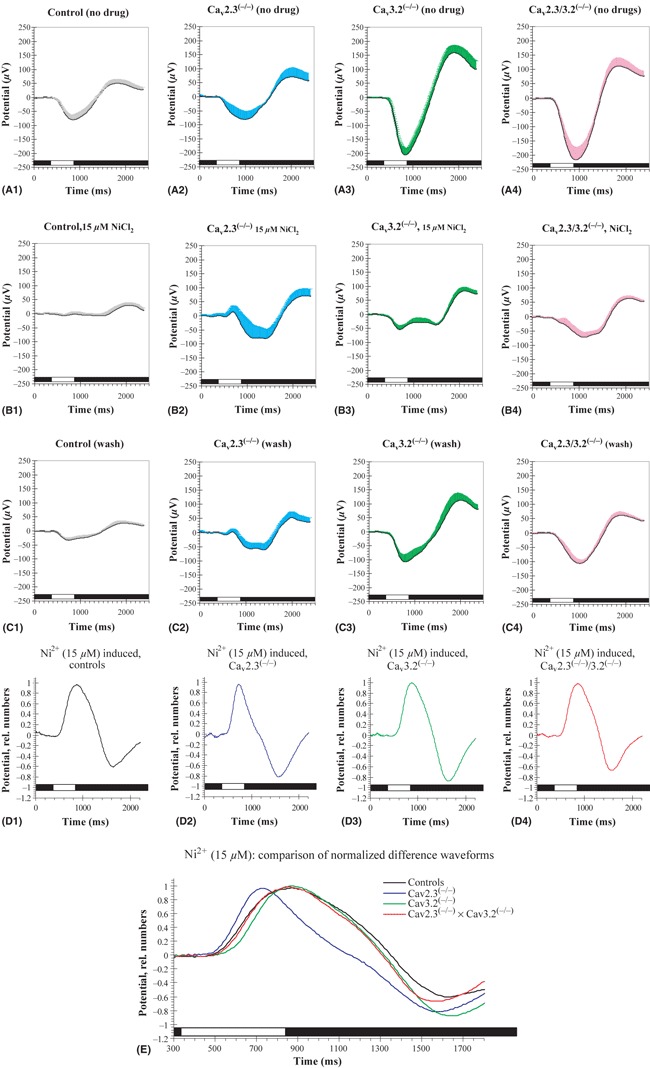
Comparison of the changes for the b-wave amplitude at low (15 μm) NiCl_2_ and after washout (short pre-equilibration of 30 min). Individual electroretinogram (ERG) traces were calculated as mean traces for control mice (panel A1–D1), for Ca_v_2.3-deficient mice (panel A2–D2), for Ca_v_3.2-deficient mice (panel A3–D3) and for double knockout mice (panel A4–D4). The upper line of panels summarizes the mean ERG traces before NiCl_2_ application (panel A1–D1), the second line of panels represents the mean traces after adding NiCl_2_ for 30 min (panel B1–B4), and the third line of panels shows the washout mean traces (panel C1–C4). In the fourth line (panel D1–D4), the differences for each genotype are plotted as normalized mean ERG trace. The flash of light (as in all other experiments for 0.5 second at 63 mlx) is indicated by the white bar. In panel (E), the four normalized ERG traces from the D-panel line are superimposed for the shorter time interval as indicated.

For a more systematic comparison between control mice and mice lacking either Ca_v_2.3 or Ca_v_3.2 or both Ni^2+^-sensitive Ca^2+^ channels, the corresponding ERG traces after 30-min NiCl_2_ superfusion (Fig. 4B1–B4) and after washout (Fig. 4C1–C4) were determined as mean values from several independent experiments (*n* = 4–8). The mean ERGs after NiCl_2_ superfusion (Fig. 4B1–B4) were subtracted from the mean ERGs before NiCl_2_ superfusion (Fig. 4A1–A4). As a result, these difference waveforms were normalized for each genotype and plotted as Fig. 4D1–D4. The overlay of these normalized waveform traces best reveals the major differences seen when one of the two NiCl_2_-sensitive Ca^2+^ channels is missing. In Ca_v_2.3-deficient mice, a later part of the b-wave component is missing, while in Ca_v_3.2-deficient mice an earlier part is absent (Fig. 4E), suggesting that both Ni^2+^-sensitive channels may contribute to transretinal signalling during the Ni^2+^-induced increase in the b-wave amplitude.

**Fig. 5:**
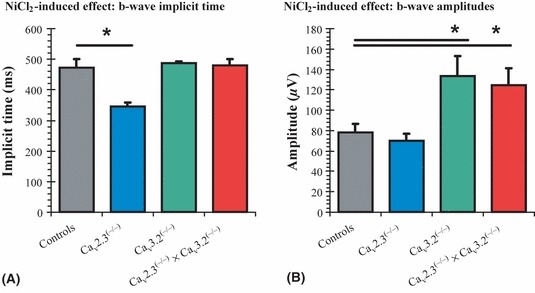
Summary of implicit times and mean amplitudes for the murine electroretinogram (ERG) b-wave at low (15 μm) NiCl_2_ (short pre-equilibration of 30 min). (A) Implicit times for the four genotypes as indicated are calculated from the normalized mean ERG traces as shown in Fig. 4D1–D4. (B) Mean b-wave amplitudes for the four genotypes as indicated are calculated from the difference traces as shown in Fig. 4C1–C4. *Significant differences (Student’s *t*-test, p < 0.05) are labelled by an asterisk.

The normalized Ni^2+^-induced ERGs (subtracted values) were compared for the four genotypes by its implicit times (Fig. 5A). The mean implicit time of the normalized b-wave amplitude was 474 ± 27 ms (*n* = 5 retinas) for control mice and was significantly reduced in Ca_v_2.3-deficient mice to 346 ± 12 ms (*n* = 8; p < 0.001), confirming the observation from the visual inspection of the overlay traces (Fig. 4E). The corresponding values for the Ca_v_3.2-deficient mice and the double knockout animals were 488 ± 4 ms (*n* = 4) and 480 ± 21 ms (*n* = 6), respectively, and did not differ significantly from control mice, suggesting that at least in Ca_v_2.3-deficient mice a major component of the b-wave is missing, which in control animals causes the delayed depolarization during retinal signalling.

Before normalization, the amplitude size of the Ni^2+^-induced component of the b-wave was also compared between the four genotypes. It is significantly increased in Ca_v_3.2-deficient mice (134 ± 20 μV; *n* = 5) and double knockout animals (125 ± 16 μV; *n* = 4) compared to controls (79 ± 8 μV; *n* = 6) and Ca_v_2.3-deficient mice (70 ± 7 μV *n* = 14) (Fig. 5B), suggesting that the lack of the Ni^2+^-sensitive Ca_v_3.2 Ca^2+^ channel also severely influences transretinal signalling, but differently than the mechanism in the Ca_v_2.3-triggered pathway. Together these results indicate that during the postsynaptic depolarization, which is described by the shape of the b-wave, two different processes exist. The Ca_v_2.3-triggered processes may rather be involved in later steps during postsynaptic depolarization, while Ca_v_3.2 may be involved in earlier steps.

## Discussion

Similar to other regions of the nervous system, the Ca^2+^-triggered neurotransmitter release in vertebrate photoreceptors and bipolar cells of the retina is mediated by high- and low-voltage-gated Ca^2+^channels (Wilkinson & Barnes 1996; Pan 2000; Pan et al. 2001). L-type voltage-gated Ca^2+^ channels control the tonic glutamate release from retinal photoreceptors and bipolar cells. At least three different types of high-voltage-activated L-type Ca^2+^ channels are expressed in the retina including Ca_v_1.2, Ca_v_1.3 and Ca_v_1.4. Additional voltage-gated Ca^2+^ channels are localized in the inner plexiform layers of the vertebrate retina (Kamphuis & Hendriksen 1998; Xu et al. 2002), which are important for multiple steps during retinal signal processing.

Ni^2+^-sensitive retinal signalling can be recorded as the increase in the ERG b-wave amplitude after superfusion of low (15 μm) NiCl_2_ solutions. For the bovine retina, a similar amplitude increase was observed when superfusing ZnCl_2_ (Siapich et al. 2010), which represents the more physiological cation. For the bovine retina Ni^2+^-mediated and GABAergic feedback signalling was identified to be mediated mainly through GABA-A and GABA-C receptors (Siapich et al. 2009). As the effect of GABA-A- and GABA-C-receptor antagonists was only slightly larger (1.9-fold increase in the b-wave amplitude) compared to the NiCl_2_-mediated increase (1.5-fold) (Lüke et al. 2005a), one may assume that the role of Ni^2+^-sensitive voltage-gated Ca^2+^ channels contributes mainly to this effect. In the studies with bovine retina, the pharmacoresistant R-type voltage-gated Ca^2+^ channel was assumed to be involved in this GABAergic signal transduction. But the Ni^2+^-sensitive Ca_v_3.2 T-type voltage-gated Ca^2+^ channels show an identical high affinity towards this divalent heavy metal cation. Therefore, both mouse models lacking either Ca_v_2.3 or Ca_v_3.2 or both Ca^2+^ channels were analysed more in detail.

During the course of these experiments, it was observed for the isolated bovine retina (Siapich et al. 2009) as well as for the murine retina that the capability of the Ni^2+^-mediated stimulation was lost faster than the basal transretinal signalling. The slow but continuous increase in the b-wave amplitude must be interpreted as a continuous loss of GABAergic inhibition onto ON-bipolar cells, which mainly contribute by their light-evoked depolarization to the ERG b-wave. It was difficult to standardize this slow reduction of physiological inhibition. Therefore, any preincubation of the isolated retina was shortened to a minimum, just to reach a reliable starting condition for ERG recording. To calculate the ‘gain’ of Ni^2+^-mediated b-wave increase, the maximum difference in amplitude was compared between four genotypes, and the normalized shape of the ERG was analysed and compared as a difference in implicit time. All these precautions were necessary to get a reproducible way of experimental work for answering three main questions. First, does Ca_v_2.3 in mice contribute to the well-described NiCl_2_-mediated increase in the ERG b-wave amplitude as observed from the isolated and superfused bovine retina (Lüke et al. 2005a; Siapich et al. 2009)? Second, is Ca_v_2.3 the only highly NiCl_2_-sensitive voltage-gated Ca^2+^ channel involved in the increase in the b-wave amplitude, which may be caused by a loss of GABAergic feedback regulation? And finally, could additional NiCl_2_-sensitive targets be involved in retinal signalling?

To answer question one, the present report shows that the gene inactivation of Ca_v_2.3 severely changes the ERG b-wave response. For this genotype, both kind of experiments were performed, those with and without a 4-h preincubation period. After the prolonged preincubation, the NiCl_2_-evoked b-wave increase was 1.4-fold for controls and 1.1-fold for Ca_v_2.3-deficient mice, without reaching the level of significance (except for the washout comparison). As mentioned, the accuracy was reduced after such a long 4-h preincubation period. Therefore, we switched to short pre-equilibration (30 min) and calculated differences for the NiCl_2_-mediated effects after an additional 30-min superfusion of 15 μm NiCl_2_. Even after such an application protocol, the NiCl_2_-mediated effects on the amplitude did not reach the level of significance, but the shape of the NiCl_2_-induced component was different in Ca_v_2.3-deficient mice compared with controls, leading to a significantly shortened implicit time (Fig. 5A). Together, these data implicate that Ca_v_2.3 contributes to a NiCl_2_-mediated change in retinal signalling.

To answer question two, the present data lead to the suggestion that not only Ca_v_2.3 but also Ca_v_3.2 contributes to NiCl_2_-mediated changes in retinal signalling. Most obvious is the significant effect of NiCl_2_ (15 μm) on the calculated increase in the ERG b-wave amplitude (Fig. 5B). If Ca_v_3.2 is rather responsible for an early subcomponent of the b-wave, one may expect an increase in the implicit time for the normalized ERG b-waves, which, however, did not reach the level of significance. The large value for the calculated Ni^2+^-induced b-wave increase arises from the very large negative a-wave value before applying NiCl_2_ (see Fig. 4A3), which also holds true for the retinas from double knockout mice (Fig. 4A4), suggesting that Ca_v_3.2 T-type Ca^2+^ channels may prevent a maximum hyperpolarization in retinas from controls and still in Ca_v_2.3-deficient mice before any application of NiCl_2_. Consecutively, the NiCl_2_-meditated increase in the ERG b-wave amplitude is always larger in Ca_v_3.2-deficient mice. Together, these results indicate that besides Ca_v_2.3, Ca_v_3.2 also contributes to retinal signalling, when analysed by the superfusion of NiCl_2_. Both voltage-gated Ca^2+^ channels share structural homology within the high-affinity Ni^2+^-binding site (Lee et al. 1999), and both channels contain critical histidine residues that are responsible for the high-affinity Ni^2+^ interaction (Kang et al. 2006, 2007).

To answer the last question, one must pay attention to the NiCl_2_-mediated increase in the double knockout mice. The ERGs recorded from this genotype also reveal a NiCl_2_-mediated increase, which is slightly but not significantly smaller than in Ca_v_3.2-deficient mice but still larger than in both other genotypes (Fig. 5B). Interestingly, the implicit time, which was significantly shortened in Ca_v_2.3- and slightly but not significantly increased in Ca_v_3.2-deficient mice, returns to a value similar to control retinas (Fig. 5A). These results clearly imply that additional Ni^2+^-sensitive targets besides both highly NiCl_2_-sensitive voltage-gated Ca^2+^ channels contribute to transretinal signalling as recorded after low NiCl_2_ application. But the subtraction method helped us at least to isolate the contribution of both Ni^2+^-sensitive Ca^2+^ channel types on retinal signalling. GABA receptors themselves as well as other transporters and receptors could be involved in such reciprocal or lateral (nonreciprocal) GABAergic signalling as it was analysed for rod bipolar cells in rat (Chavez et al. 2010) and found for the bovine retina (Siapich et al. 2009).

Although not proven in the present study for mice, one might assume that the NiCl_2_-mediated responses of b-wave increase may be related to GABAergic feedback regulation as observed and described in great detail for isolated rat retina segments (Chavez et al. 2010), but further studies are needed to analyse it for the murine retina in different mouse models. In the rat study, different types of voltage-gated channels could be connected to several modes of reciprocal feedback control, including mibefradil-sensitive T-type, omega-agatoxinIVA-sensitive P-/Q-type and Ni^2+^-sensitive R- and T-type Ca^2+^ channels (Chavez et al. 2010).

Our present study led to the identification of an earlier and a later Ni^2+^-sensitive component of the b-wave retina response. For the goldfish (*Carassius auratus*) retina, the temporal characteristics of reciprocal signalling were analysed recently more in detail (Vigh & Von Gersdorff 2005). While the released glutamate from bipolar cell terminals first activates AMPA receptors, followed by a fast and transient GABA-A-mediated feedback, the subsequent prolonged activation via NMDA receptors triggers also a slow, sustained GABA-C-mediated reciprocal inhibition. For bovine retina, antagonists for both ionotropic GABA receptor types were shown to participate in the b-wave increase, mediated by Ni^2+^-sensitive voltage-gated Ca^2+^ channels. In conclusion, the shift of the difference wave forms for Ca_v_2.3-deficient ERGs to a shorter implicit time may be caused by omitting the GABA effect onto GABA-C receptors. Further, the shift of the b-wave amplitude for Ca_v_3.2-deficient mice may rather point to a lack of signalling to GABA-A receptors on bipolar neurons, which still has to be proven in consecutive studies.

Together, the results from our study and the results reported in the literature provide strong evidence that low NiCl_2_ concentrations (15 μm) may change the ERG b-wave response by two different voltage-gated Ca^2+^ channels and also by additional highly Ni^2+^-sensitive targets. While Ni^2+^ cations may present nonphysiological cations, future studies may include the more physiological effects of Zn^2+^, which is highly concentrated in the vertebrate retina and reveals pronounced effects on the isolated bovine retina (Siapich et al. 2010). Future studies with the isolated vertebrate retina may also help to elucidate in more detail the mechanisms of the inhibitory reciprocal and nonreciprocal signalling, which leads to a better understanding in more complicated neuronal circuits as found in hippocampus (Weiergräber et al. 2006) and the thalamocortical neurons (Weiergraber et al. 2010).
